# Syk/Src Pathway-Targeted Inhibition of Skin Inflammatory Responses by Carnosic Acid

**DOI:** 10.1155/2012/781375

**Published:** 2012-04-10

**Authors:** Jueun Oh, Tao Yu, Soo Jeong Choi, Yanyan Yang, Heung Soo Baek, Soon Ae An, Lee Kyoung Kwon, Jinsol Kim, Ho Sik Rho, Song Seok Shin, Wahn Soo Choi, Sungyoul Hong, Jae Youl Cho

**Affiliations:** ^1^Department of Genetic Engineering, Sungkyunkwan University, Suwon 440-746, Republic of Korea; ^2^Medical Beauty Research Institute, AmorePacific R&D Center, Yongin 446-729, Republic of Korea; ^3^Department of Immunology and Physiology, College of Medicine, Konkuk University, Chungju 380-701, Republic of Korea

## Abstract

Carnosic acid (CA) is a diterpene compound exhibiting antioxidative, anticancer, anti-angiogenic, anti-inflammatory, anti-metabolic disorder, and hepatoprotective and neuroprotective activities. In this study, the effect of CA on various skin inflammatory responses and its inhibitory mechanism were examined. CA strongly suppressed the production of IL-6, IL-8, and MCP-1 from keratinocyte HaCaT cells stimulated with sodium lauryl sulfate (SLS) and retinoic acid (RA). In addition, CA blocked the release of nitric oxide (NO), tumor necrosis factor (TNF)-*α*, and prostaglandin E_2_ (PGE_2_) from RAW264.7 cells activated by the toll-like receptor (TLR)-2 ligands, Gram-positive bacterium-derived peptidoglycan (PGN) and pam3CSK, and the TLR4 ligand, Gram-negative bacterium-derived lipopolysaccharide (LPS). CA arrested the growth of dermatitis-inducing Gram-positive and Gram-negative microorganisms such *Propionibacterium acnes, Pseudomonas aeruginosa*, and *Staphylococcus aureus*. CA also blocked the nuclear translocation of nuclear factor (NF)-*κ*B and its upstream signaling including Syk/Src, phosphoinositide 3-kinase (PI3K), Akt, inhibitor of *κ*B*α* (I*κ*B*α*) kinase (IKK), and I*κ*B*α* for NF-*κ*B activation. Kinase assays revealed that Syk could be direct enzymatic target of CA in its anti-inflammatory action. Therefore, our data strongly suggest the potential of CA as an anti-inflammatory drug against skin inflammatory responses with Src/NF-*κ*B inhibitory properties.

## 1. Introduction

Skin inflammation (dermatitis) includes many inflammatory symptoms occurring in the skin layer in the form of a rash [1]. Infections with Gram-positive and Gram-negative bacteria (e.g., *Propionibacterium acnes*, *Pseudomonas aeruginosa*, and *Staphylococcus aureus) *and fungi (*Candida albicans *ATCC 10231, and *Aspergillus niger*), exposure to irritating chemicals such as sodium lauryl sulfate (SLS) or retinoic acid (RA), and various allergens, such as 2,4-dinitrophenol (DNP), are regarded as major inflammatory signaling inducers causing skin rash [2–4]. Although most skin inflammatory symptoms are not serious, some can lead to life-threatening conditions such as meningitis or severe allergic reaction including anaphylaxis and allergic purpura [5, 6]. In addition, the undesirable cosmetic appearance often associated with inflammation of the facial skin underscores the importance of developing a promising agent to rapidly ameliorate such skin symptoms. When inflammatory signals occur in epithelial cells, macrophages, keratinocytes, mast cells, and Langerhans cells of the skin layer, various inflammatory mediators, including the interleukin (IL)-1, IL-6, and tumor necrosis factor (TNF)-*α* cytokines, IL-8 and monocyte chemotactic protein (MCP)-1 chemokines, and nitric oxide (NO) and prostaglandin E_2_ (PGE_2_) mediators, are produced [7, 8]. For these events, toll-like receptor (TLR)- 2 or TLR4-dependent activation receptors [9, 10] or independent stimulation of intracellular signaling cascades composed of Src and syk nonreceptor protein tyrosine kinases, phosphoinositide- 3-kinase (PI3K), phosphoinositide-dependent kinase 1 (PDK1), and Akt (protein kinase B) serine-threonine protein kinases, as well as the activation and upregulation of nuclear factor (NF)-*κ*B and activator protein (AP)-1 transcription factors [11, 12], transcriptionally activate inflammatory cells to express numerous inflammatory genes encoding pro-tumor necrosis factor (TNF)-*α* for TNF-*α* secretion, inducible NO synthase (iNOS) for NO release, and cyclooxygenase (COX)-2 for prostaglandin E_2_ (PGE_2_) production [13–16].

**Figure 1:**
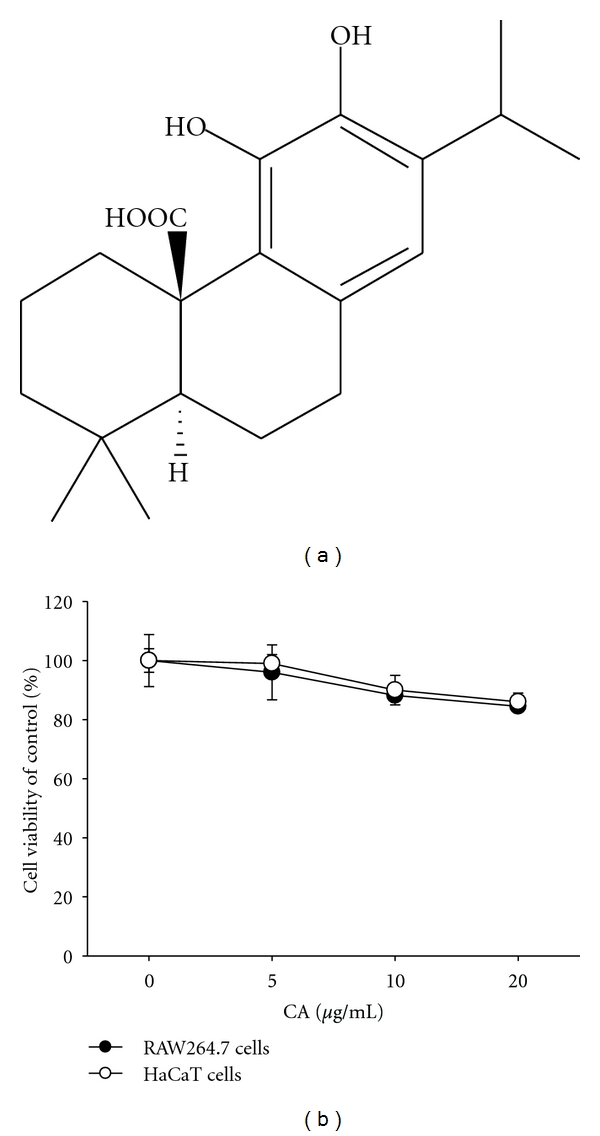
The chemical structure of CA and its cytotoxic activity. (a) Chemical structure of CA. (b) The viability of RAW264.7 and HaCaT cells was determined by MTT assays.

Carnosic acid (CA; Figure 1(a)), isolated from the fresh leaves of* Rosmarinus officinalis *L. [17], is a pro-electrophilic diterpene displaying multiple actions similar to other diterpenoid compounds such as carnosol [18]. This compound has been reported to possess antioxidative, anti-cancer, anti-angiogenic, anti-inflammatory, anti-metabolic disorder, photoprotective, and hepatoprotective and neuroprotective activities [19–21]. How this compound can have multi-potential pharmacological properties is not yet well understood. Although the molecular target of CA has not been fully identified, its antioxidative activity, activation of peroxisome proliferator-activated receptor gamma, and 5-lipoxygenase inhibition are regarded as major mechanisms of its multifunctional pharmacology [22–24].

Although polyphenolic compounds including CA are currently being investigated for industrial applications, their mass production by chemical syntheses and their photostability remain critical factors for consideration. CA is chemically stable and CA production and yield have been greatly improved by current synthetic methods [25, 26]. In addition, because numerous cosmetic and pharmaceutical companies focus on skin inflammation symptoms, we were encouraged to study the effect of CA on skin inflammation and its anti-inflammatory mechanism. In this study, the inhibitory activity of CA on the production of inflammatory mediators in macrophages and keratinocytes and the inhibitory target molecule(s) of CA were examined. 

## 2. Materials and Methods

### 2.1. Materials

CA (95% purity), phenoxyethanol, octanediol, methylparaben, magnotics, ampicillin, 3-(4,5-dimethylthiazol-2-yl)-2,5-diphenyltetrazolium bromide (MTT), peptidoglycan (PGN), and lipopolysaccharide (LPS; *E. coli* 0111:B4) were purchased from Sigma Chemical Co. (St. Louis, MO, USA). Piceatannol (picea) and PP2 were obtained from Calbiochem (La Jolla, CA, USA). Luciferase constructs containing promoters sensitive to NF-*κ*B, CREB, and AP-1 were gifts from Profs. Chung Hae Young (Pusan National University, Pusan, Republic of Korea) and Man Hee Rhee (Kyungpook National University, Daegu, Republic of Korea). Enzyme immunoassay (EIA) kits and enzyme-linked immunosorbent assay (ELISA) kits for determining PGE_2_, IL-6, IL-8, MCP-1, and TNF-*α* were purchased from Amersham (Little Chalfont, Buckinghamshire, UK). Fetal bovine serum and RPMI1640 were obtained from Gibco (Grand Island, NY, USA). The murine macrophage cell line, RAW264.7, the human keratinocyte cell line, HaCaT, the rat basophilic leukemia mast cell line, RBL-2H3, and the human embryonic kidney cell line, HEK293, were purchased from the ATCC (Rockville, MD, USA). All other chemicals were of analytical grade and were obtained from Sigma. Phosphospecific or total antibodies to p65, p50, Src, Syk, PDK1, p85, Akt, I*κ*B*α*, lamin A/C, and *β*-actin were obtained from Cell Signaling (Beverly, MA, USA).

### 2.2. Cell Culture

RAW264.7, HaCaT, RBL-2H3, and HEK293 cells were cultured in DMEM or RPMI1640 medium supplemented with 10% heat-inactivated fetal bovine serum (FBS; Gibco, Grand Island, NY, USA), glutamine, and antibiotics (penicillin and streptomycin) at 37°C under 5% CO_2_. For each experiment, cells were detached with a cell scraper. At the cell density used for the experiments (2×10^6^ cells/mL), the proportion of dead cells was less than 1% as measured by Trypan blue dye exclusion.

### 2.3. Cell Viability Test

After preincubation of RAW264.7 and HaCaT cells (1 × 10^6^ cells/mL) for 18 h, CA (0 to 20 *μ*g/mL) was added to the cells and incubated for 24 h. The cytotoxic effect of CA was then evaluated by a conventional MTT assay, as reported previously [27, 28]. At 3 h prior to culture termination, 10 *μ*L MTT solution (10 mg/mL in phosphate buffered-saline, pH 7.4) was added to each well, and the cells were continuously cultured until termination of the experiment. The incubation was halted by the addition of 15% sodium dodecyl sulfate (SDS) into each well, solubilizing the formazan [29]. Absorbance at 570 nm (OD_570–630_) was measured using a SpectraMax 250 microplate reader.

### 2.4. Determination of NO, PGE_2_, IL-6, IL-8, MCP-1, and TNF-*α*

After preincubation of RAW264.7 and HaCaT cells (1 × 10^6^ cells/mL) for 18 h, cells were pretreated with CA (0 to 20 *μ*g/mL) for 30 min and further incubated with LPS (1 *μ*g/mL), pam3CSK (10 *μ*g/mL), or PGN (10 *μ*g/mL) for 6 (TNF-*α*) or 24 (all other treatments) h. The inhibitory effect of CA on the production of NO, PGE_2_, IL-6, IL-8, MCP-1, and TNF-*α* was determined by analyzing NO, PGE_2_, IL-6, IL-8, MCP-1, and TNF-*α* levels with Griess reagent and enzyme-linked immunosorbent assay (ELISA) kits as described previously [30, 31]. 

### 2.5. *β*-Hexosaminidase Secretion Assay

RBL-2H3 cells were grown in DMEM supplemented with 10% FBS in a 5% CO_2_ atmosphere. RBL-2H3 cells (2 × 10^5^ cells/well) pretreated with CA were sensitized with 1 *μ*g/mL anti-DNP IgE for 1 h at 37°C. IgE-sensitized cells were washed with PBS and stimulated with 4 *μ*g/mL 2,4-dinitrophenylated bovine serum albumin (DNP-BSA) in PBS at 37°C for 30 min. Degranulation was assessed by measuring *β*-hexosaminidase release. Briefly, 40 *μ*L supernatant and 100 *μ*L 2 mM *p*-nitrophenyl-*N-*acetyl-*β*-D-glucosaminide (in 0.4 M citrate and 0.2 M phosphate buffer, pH 4.5) were added to each well of a 96-well plate, and color was developed for 30 min at 37°C. The enzymatic reaction was terminated by adding 200 *μ*L 0.2 M glycine-NaOH, pH 10.7. The absorbance at 405 nm was measured using a SpectraMax 250 microplate reader.

### 2.6. mRNA Analysis by Semiquantitative Reverse Transcriptase Polymerase Chain Reaction (RT-PCR)

To determine cytokine mRNA expression levels, total RNA was isolated from LPS-treated RAW264.7 cells using TRIzol Reagent (Gibco BRL) according to the manufacturer's instructions. Total RNA was stored at −70°C until use. Semiquantitative RT reactions were conducted as reported previously [32, 33]. The primers (Bioneer, Daejeon, Republic of Korea) used are listed in Table 1.

### 2.7. Luciferase Reporter Gene Activity Assay

HEK293 cells (1 × 10^6^ cells/mL) were transfected with 1 *μ*g plasmid containing NF-*κ*B-Luc or AP-1-Luc along with *β*-galactosidase using the calcium phosphate method in a 12-well plate according to the manufacturer's protocol [34]. The cells were used for experiments 48 h after transfection. Luciferase assays were performed using the Luciferase Assay System (Promega) as reported previously [35].

### 2.8. Preparation of Cell Lysates and Nuclear Fraction, Immunoblotting, and Immunoprecipitation

RAW264.7 cells (5 × 10^6^ cells/mL) were washed three times in cold PBS with 1 mM sodium orthovanadate and lysed by a sonicator in lysis buffer (20 mM Tris-HCl, pH 7.4, 2 mM EDTA, 2 mM ethylene glycol tetraacetic acid, 50 mM *β*-glycerophosphate, 1 mM sodium orthovanadate, 1 mM dithiothreitol, 1% Triton X-100, 10% glycerol, 10 *μ*g/mL aprotinin, 10 *μ*g/mL pepstatin, 1 mM benzamide, and 2 mM PMSF) for 30 min with rotation at 4°C. The lysates were clarified by centrifugation at 16,000 ×g for 10 min at 4°C and stored at −20°C until needed.

Nuclear lysates were prepared in a three-step procedure [36]. After treatment, cells were collected with a rubber policeman, washed with PBS, and lysed in 500 *μ*L lysis buffer containing 50 mM KCl, 0.5% Nonidet P-40, 25 mM HEPES (pH 7.8), 1 mM phenylmethylsulfonyl fluoride, 10 *μ*g/mL leupeptin, 20 *μ*g/mL aprotinin, and 100 *μ*M 1,4-dithiothreitol (DTT) on ice for 4 min. Cell lysates were then centrifuged at 19,326 ×g for 1 min in a microcentrifuge. In the second step, the nuclear fraction pellet was washed once in washing buffer, which was the same as the lysis buffer without Nonidet P-40. In the final step, nuclei were treated with an extraction buffer containing 500 mM KCl, 10% glycerol, and the other reagents in the lysis buffer. The nuclei/extraction buffer mixture was frozen at −80°C and then thawed on ice and centrifuged at 19,326 ×g for 5 min. The supernatant was collected as a nuclear extract. Soluble cell lysates were immunoblotted, and protein levels were visualized as previously reported [37]. For immunoprecipitation, cell lysates containing equal amounts of protein (500 *μ*g) from RAW264.7 cells (1 × 10^7^ cells/mL) treated with or without LPS (1 *μ*g/mL) for 2.5 min were precleared with 10 *μ*L protein A-coupled Sepharose beads (50% v/v) (Amersham, UK) for 1 h at 4°C. Pre-cleared samples were incubated with 5 *μ*L anti-JAK2 antibody overnight at 4°C. Immune complexes were mixed with 10 *μ*L protein A-coupled Sepharose beads (50% v.v.) and rotated for 3 h at 4°C.

### 2.9. Enzyme Assay

For evaluating the inhibition of Src and Syk kinase activity using purified enzymes, the kinase profiler service from Millipore (Billerica, MA, USA) was used. In a final reaction volume of 25 *μ*L, purified enzymes (1–5 mU) were incubated with the reaction buffer. The reaction was initiated by the addition of MgATP. After incubation for 40 min at room temperature, the reaction was stopped by the addition of 5 mL 3% phosphoric acid. Ten microliters of the reaction mixture was then spotted onto a P30 filtermat and washed three times for 5 min in 75 mM phosphoric acid and once in methanol prior to drying and scintillation counting.

### 2.10. In Vitro Antimicrobial Activity Assay

The minimum inhibitory concentration (MIC, *μ*g/mL) of the fourteen synthesized compounds was determined under conditions described in the literature [38] for each assay against *Propionibacterium acnes *(ATCC 6919), *Staphylococcus aureus* (ATCC 33592),* Escherichia coli *(ATCC 25922)*, Candida albicans *(ATCC 10231), and* Aspergillus niger *(ATCC 9142).

### 2.11. Statistical Analysis

**Figure 2:**
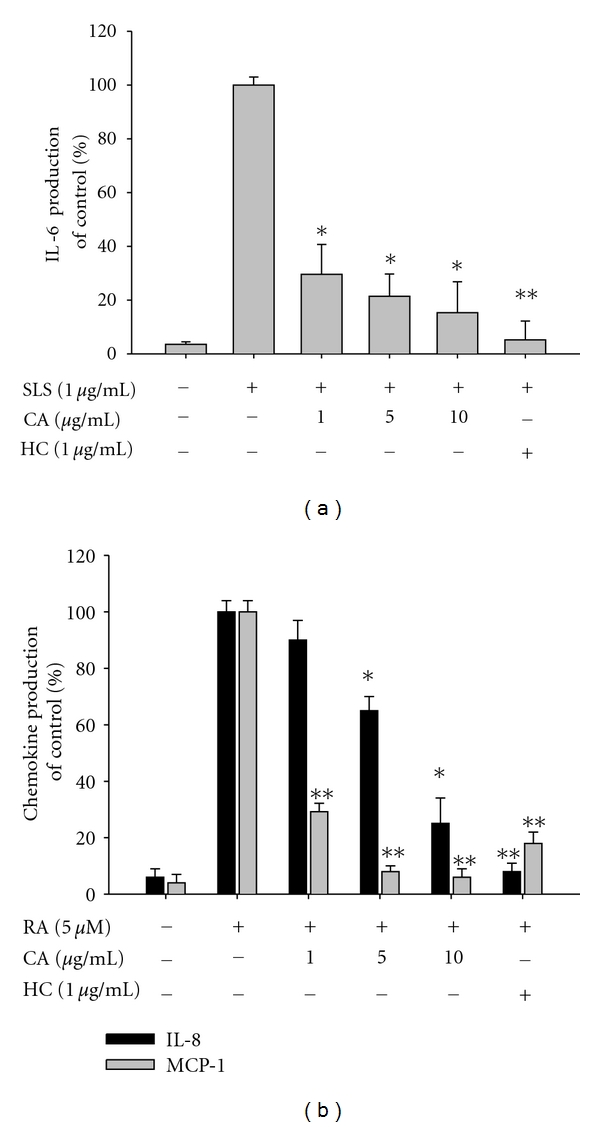
Effect of CA on the production of inflammatory cytokines and chemokines in HaCaT cells stimulated with SLS and RA. (a) and (b) Levels of IL-6, IL-8, and MCP-1 were determined by ELISA from culture supernatants of HaCaT cells treated with CA (0 to 10 *μ*g/mL) or hydrocortisone (HC, 1 *μ*g/mL) in the presence or absence of SLS (1 *μ*g/mL) or RA (5 *μ*M) for 24 h. *P < 0.05 and **P < 0.01 compared to the control.

**Figure 3:**
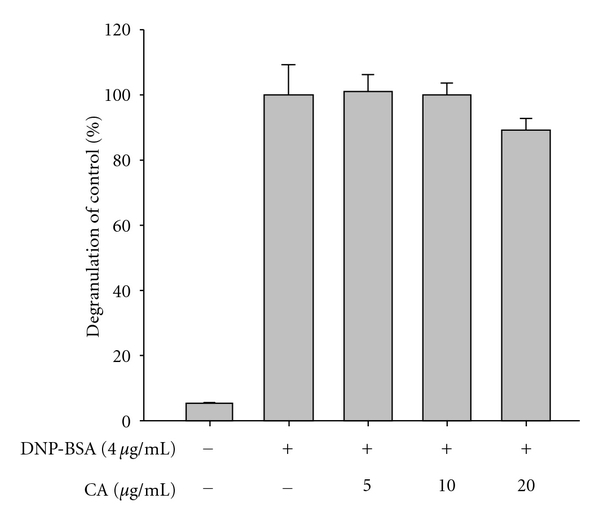
Effect of CA on the degranulation of IgE-sensitized RBL-2H3 cells treated with DNP-BSA. IgE-sensitized RBL-2H3 cells (2 × 10^5^ cells/mL) were incubated with CA in the presence or absence of DNP-BSA (4 *μ*g/mL) for 6 h. Degranulation was determined by measuring *β*-hexosaminidase activity.

**Figure 4:**
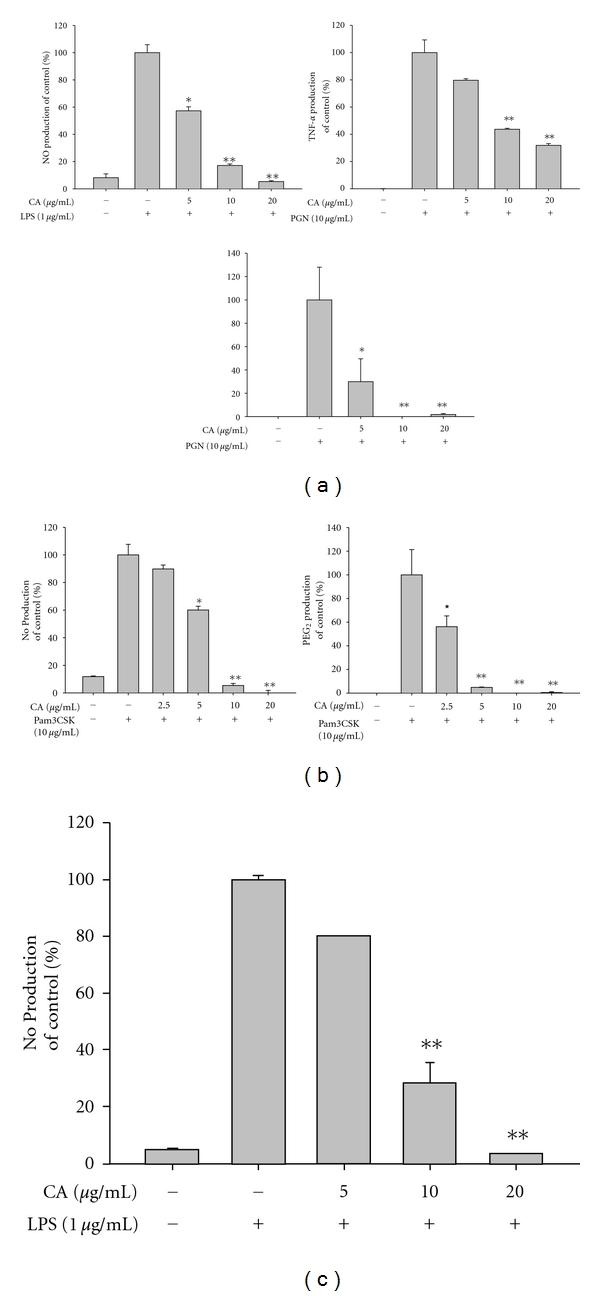
Effect of CA on the production of NO, TNF-*α*, and PGE_2_, in activated RAW264.7 cells. (a), (b), and (c) Levels of NO, TNF-*α*, and PGE_2_ were determined by the Griess assay, ELISA, and EIA, respectively, from RAW264.7 cell culture supernatants treated with CA (0 to 20 *μ*g/mL) in the presence or absence of peptidoglycan (PGN, 10 *μ*g/mL) (a), pam3CSK (10 *μ*g/mL) (b), or LPS (1 *μ*g/mL) (c), for 6 h (TNF-*α*) or 24 h (NO and PGE_2_). *P < 0.05 and **P < 0.01 compared to the control.

**Figure 5:**
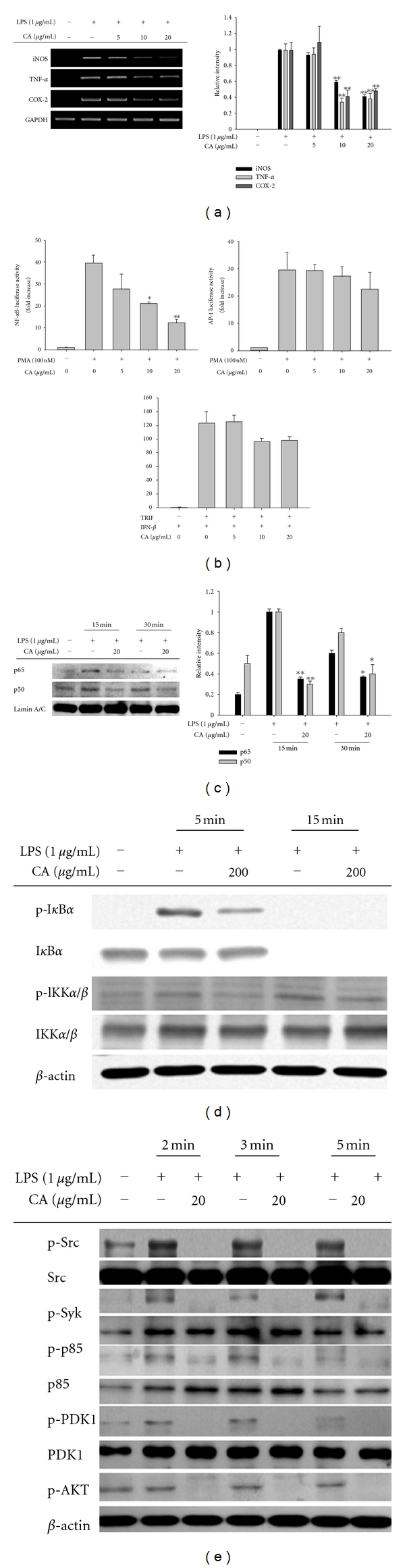
Effect of CA on the mRNA expression of proinflammatory genes, the activation of transcription factors, and upstream signaling cascades for NF-*κ*B activation. (a) The mRNA levels (left panel) of genes encoding iNOS, TNF-*α*, and COX-2 were determined by semiquantitative PCR. (b) HEK293 cells cotransfected with NF-*κ*B-Luc, IFN-*β*-promoter-Luc, or AP-1-Luc plasmid constructs (1 *μ*g/mL each) and *β*-gal (as a transfection control) were treated with CA (0 to 20 *μ*g/mL) in the presence or absence of PMA (100 nM) or by cotransfection with the adapter molecule, TRIF. Luciferase activity was measured using a luminometer. (b) Levels of NF-*κ*B family proteins, p50 and p65, in the nuclear fraction were determined by immunoblotting analyses using antibodies against total protein. (d) and (e) Phosphoprotein or total protein levels of I*κ*B*α*, IKK, Akt, PDK1, p85, Src, Syk, and *β*-actin from cell lysates were determined by immunoblotting analyses using phosphospecific or total protein antibodies. Relative intensity was calculated by densitometric scanning. *P < 0.05 and **P < 0.01 compared to the control.

**Figure 6:**
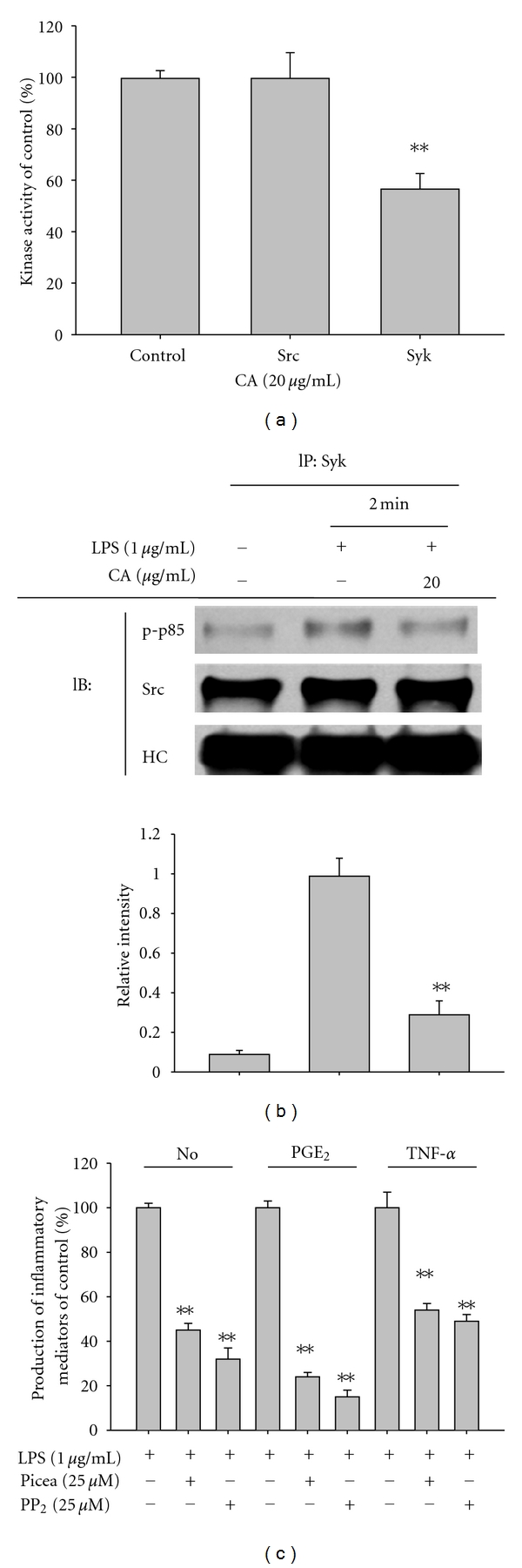
Involvement of the Syk and Src pathways as a target of the CA-mediated anti-inflammatory response. (a) Kinase activities of Syk and Src were determined by a direct kinase assay using purified enzymes. The control was set at 100% with each enzyme (Src or Syk) activity obtained only with vehicle treatment. (b) RAW264.7 cells (5 × 10^6^ cells/mL) were incubated with CA (20 *μ*g/mL) in the presence or absence of LPS (1 *μ*g/mL) for 2 min. After preparing total lysates, levels of phospho (p)-p85 binding to Syk (left panel) or Src (right panel) were identified by immunoprecipitation with antibodies to Syk or Src and immunoblotting with antibodies to rabbit immunoglobulin heavy chain, Syk, Src, and p-p85. (C) Culture supernatants prepared from LPS-treated RAW264.7 cells pretreated with standard Src and Syk inhibitors (PP2 and piceatannol (Picea)) were assayed for NO, TNF-*α*, and PGE_2_. Relative intensity of each blot was calculated from densitometric scanning. HC: heavy chain; **P < 0.01 compared to the control.

Data (Figures 1(b), 2, 3, 4, 5(b), 6(a), and 6(c)) are expressed as the mean ± standard deviation (SD) as calculated from one (*n* = 6) of two independent experiments. Other data are representative of three different experiments with similar results. For statistical comparisons, results were analyzed using analysis of variance/Scheffe's posthoc test and the Kruskal-Wallis/Mann-Whitney test. All *P* values < 0.05 were considered statistically significant. All statistical tests were conducted using SPSS (SPSS Inc., Chicago, IL, USA).

## 3. Results and Discussion

CA is a multipotential diterpene displaying antioxidative, anticancer, antiangiogenic, anti-inflammatory, antimetabolic disorder, photoprotective, hepatoprotective, and neuroprotective activities [19–21]. Although the anti-inflammatory activity of CA has been reported previously, the molecular target of CA in its anti-inflammatory action is unknown. In addition, whether CA can block skin inflammatory responses induced by various irritants and infection with dermatological relevance has not been fully elucidated.

Our data indicate that CA up to 20 *μ*g/mL (Figure 1(a)) is nontoxic and able to reduce various inflammatory events found in the skin layer (Figure 1(b)). Thus, this compound strongly suppressed the production of cytokines induced by skin irritation brought about by the contact irritants, all-trans retinoic acid (RA) and sodium lauryl sulfate (SLS) [39]. Indeed, these chemicals dramatically enhanced the level of inflammatory cytokines and chemokines including IL-6, IL-8, and MCP-1 secreted from keratinocytic HaCaT cells. Interestingly, CA (0 to 10 *μ*g/mL) remarkably reduced the production of these molecules to basal levels (Figures 2(a) and 2(b)). Hydrocortisone also displayed strong inhibitory activity on the production of cytokines and chemokines (Figures 2(a) and 2(b)), as reported previously [40], indicating that the experimental conditions were well established. To test whether CA can diminish itching of the skin based on histamine release [41], CA was examined using mast cells stimulated with anti-DNP-IgE. As seen in Figure 3, anti-DNP-IgE treatment stimulated the release of histamine up to 20-fold as assessed by measuring *β*-hexosaminidase activity [42]. CA did not block histamine release, implying that this compound is not able to attenuate the skin itching component of the inflammatory response.

Most serious skin inflammation is caused by infection with various microorganisms including the Gram-positive *Propionibacterium acnes* and *Staphylococcus aureus* and the Gram-negative *Pseudomonas aeruginosa* [43, 44]. Therefore, the ability of CA to modulate bacterium-induced inflammatory responses and to directly kill these bacteria was investigated. First, the anti-inflammatory activity of CA was examined using peptidoglycan, a major component of the cell wall of Gram-positive bacteria, as a TLR2 ligand [45]. Intriguingly, CA clearly reduced the release of NO, PGE_2_, and TNF-*α* triggered by PGN (10 *μ*g/mL; Figure 4(a)). Consistent with this finding, the pam3CSK-induced production of NO and PGE_2_ was also similarly suppressed by CA exposure (Figure 4(b)), suggesting that CA can prevent *P. acnes-*mediated inflammation. In addition, CA showed a similar pattern of inhibition of NO production stimulated by LPS (Figure 4(c)), as was seen in LPS-treated microglial cells [21]. Of these inflammatory mediators, CA blocked PGE_2_ production more potently than NO and TNF-*α* in macrophage-like RAW264.7 cells. This suggests that PGE_2_ could be a strong target for CA-mediated anti-inflammatory activity, as demonstrated by the pharmacology of various anti-inflammatory drugs and agents such as resveratrol, quercetin, and curcumin [46]. Surprisingly, CA also inhibited the growth of *P. acnes* with an MIC value of 19.5 *μ*g/mL. A representative antibiotic, ampicillin (MIC = 2 *μ*g/mL), but not the recently developed antibiotic herb, Magnotics, which is known to inhibit pimples [47], showed much stronger activity (Table 2). Furthermore, CA also suppressed the growth of several microorganisms including *P. aeruginosa, E. coli, S. aureus, C. albicans, *and* A. niger *with MIC values ranging from 125 to 2,000 *μ*g/mL, even though the activity was not as powerful as that of several antiseptics such as phenoxyethanol, octanediol, and methylparaben (Table 2). This implies that CA has an additional merit in that it directly kills microorganisms responsible for skin inflammation.

To understand the inhibitory mechanism by which CA suppresses the production of inflammatory mediators, the biochemical target of CA was explored using LPS-treated macrophages. The initial approach was to decide whether CA-induced inhibition of inflammatory mediator production was observed at the transcriptional or translational level by measuring mRNA levels of inflammatory genes. As expected, CA was shown to suppress the inflammatory response at the transcription level. Specifically, the mRNA levels of the genes encoding iNOS, COX-2, and TNF-*α* were remarkably reduced by CA exposure (Figure 5(a)). Two methods, a reporter gene assay (Figure 5(b)) using a construct with promoter regions binding activated NF-*κ*B, AP-1, and CREB, and a nuclear translocation level analysis of transcription factor NF-*κ*B (p50/p65) (Figure 5(c)) strongly suggested that CA could be targeted to the activation pathways for NF-*κ*B rather than AP-1 and CREB. Indeed, the upstream signaling events for NF-*κ*B activation were also clearly reduced as the phosphorylation of I*κ*B*α* and its upstream kinase IKK were reduced by CA at 5 min (Figure 5(d)). Consistent with this finding, CA treatment diminished a series of Src, Syk, p85/PI3K, PDK1, and Akt phosphorylation events for the activation of IKK that occurred between 2 and 5 min (Figure 5(e)), suggesting that the target of CA could be enzyme(s) activated at early time points in the inflammatory signaling cascade. It has been similarly reported that CA is able to block NF-*κ*B translocation in IL-1*β*-stimulated human umbilical vein endothelial cells linked to the suppression of adhesion molecule expression [48] and in TNF-*α*-stimulated human aortic smooth muscle cells linked to inhibition of cell migration and matrix metalloproteinase-9 [49]. 

It is known that the phosphorylation of Src and Syk, initially activated protein tyrosine kinases by inflammatory signaling [50, 51], is managed by their own kinase activity. Therefore, the possibility that CA can directly suppress the kinase activity of Syk or Src was confirmed by kinase assays using purified enzymes. Unexpectedly, CA (20 *μ*g/mL) only directly and partially blocked the kinase activity of Syk but not Src (Figure 6(a)). When the fact that the inhibitory activity of CA on the production of IL-6, MCP-1, NO, and PGE_2_ exhibited higher level (80 to 50%) at even 5 and 10 *μ*g/mL (Figures 2 and 4) is considered, it is strongly suggested that direct Syk inhibition is not enough to block the production of these mediators. However, the effect of CA on signaling molecule complex formation including Src or Syk examined by immunoprecipitation and immunoblotting analyses revealed that it can reduce the formation of the complex composed of active substrate phosphop85 and Syk or Src (Figure 6(b)), indicating that CA-mediated inhibition of Src and Syk phosphorylation and the activation of their downstream pathway (Figure 5(e)) could affect the generation of upstream signaling complexes, a critical event for NF-*κ*B activation [50, 51]. Finally, whether the inhibition of these enzymes by their specific inhibitors, PP2 for Src and piceatannol for Syk, showed an inhibitory pattern similar to that of CA was investigated. As Figure 6(c) depicts, the two inhibitors significantly suppressed the production of NO, PGE_2_, and TNF-*α*, as previously reported [52]. The crucial role of these enzymes in various inflammatory events was also presented previously. Thus, the activation and phosphorylation of Src and Syk in macrophages, monocytic cells, and neutrophils under inflammatory conditions have been clearly characterized [52, 53]. Furthermore, anti-inflammatory drugs such as curcumin, resveratrol, and quercetin and anti-inflammatory herbal extracts prepared from *Polygonum hydropiper, Cinnamomum aromaticum *(*cassia*)*, Sorbus commixta, *and* Sanguisorba officinalis* were shown to target these enzymes in their anti-inflammatory actions [54–56]. Therefore, our data and previous reports strongly suggest that the anti-inflammatory action of CA could be Src/Syk-targeted. 

**Figure 7:**
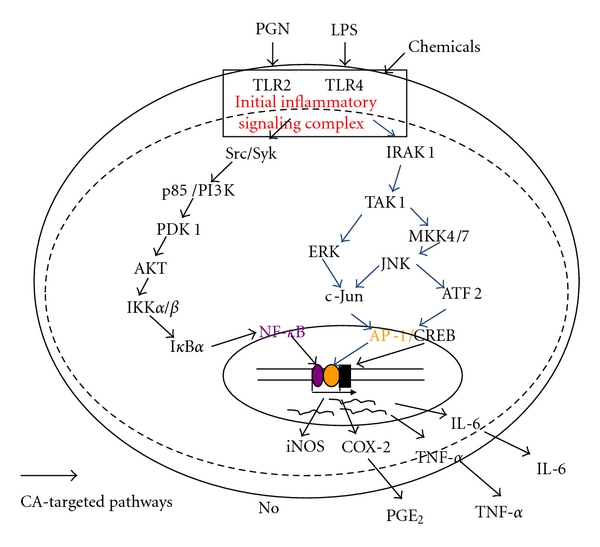
Putative inhibitory pathway of inflammatory signaling events by CA.

In summary, we have found that CA strongly blocks several skin inflammatory responses such as the production of TNF-*α*, and PGE_2_ managed by macrophages and keratinocytes. Specifically, CA was clearly diminished the activation of NF-*κ*B through the inhibition of its upstream signaling cascades composed of Syk, Src, PI3K, PDK1, Akt, IKK and I*κ*B*α* (Figure 7). Considering that it is now possible to mass-produce CA by a completely synthetic method, CA has the potential to be used as a promising anti-NF-*κ*B inhibitory drug available for skin inflammatory symptoms induced by various irritants, microorganism-derived immunogens, and allergens. To investigate this possibility, the *in vivo* efficacy using skin inflammatory models will be tested in the future. Furthermore, since two major pathways, JAK2/STAT-1 and TBK1/IRF-3 [57, 58], are also known as an important pathway for the production of inflammatory mediators in various inflammatory responses, whether CA is capable of suppressing these pathways will be continuously examined. In addition, it has been also reported that CA is able to induce Keap1/Nrf2 pathway [59] which is anti-oxidative and cytoprotective system against cellular oxidative stress [60]. Since this pathway is also known to participate in cellular anti-inflammatory responses, whether this pathway is needed in CA-mediated anti-inflammatory activity will be further confirmed in the future. 

## Figures and Tables

**Table 1:** Primer sequences used in RT-PCR analysis.

Gene	Primer sequences	
TNF-*α*	F	5′-TTGACCTCAGCGCTGAGTTG-3′
	R	5′-CCTGTAGCCCACGTCGTAGC-3′
iNOS	F	5′-CCCTTCCGAAGTTTCTGGCAGCAGC-3′
	R	5′-GGCTGTCAGAGCCTCGTGGCTTTGG-3′
COX-2	F	5′-CACTACATCCTGACCCACTT-3′
	R	5′-ATGCTCCTGCTTGAGTATGT-3′
GAPDH	F	5′-CACTCACGGCAAATTCAACGGCAC-3′
	R	5′-GACTCCACGACATACTCAGCAC-3′

**Table 2:** MIC  (*μ*g/mL) of CA and other chemicals against *P. acnes* and other microorganisms causing skin inflammation.

Compound	MIC (*μ*g/mL)					
	*P. acnes*	*P. aeruginosa*	*E. coli*	*S. aureus*	*C. albicans*	*A. niger*
CA	19.5	2000	2000	500	1000	125
Phenoxyethanol		4000	4000	6500	4000	2500
Octanediol		5000	1250	5000	5000	1250
Methylparaben		1200–1400	1400	1400	1400	1000
Magnotics	128	NT	NT	NT	NT	NT
Ampicillin	2	NT	NT	NT	NT	NT

NT: not tested.
